# Recommendations for streamlining precision medicine in breast cancer care in Latin America

**DOI:** 10.1002/cnr2.1400

**Published:** 2021-05-03

**Authors:** Isabel Alvarado‐Cabrero, Franco Doimi, Virginia Ortega, Jurema Telles de Oliveira Lima, Rubén Torres, Lilian Torregrosa

**Affiliations:** ^1^ Department of Pathology Mexican Oncology Hospital, IMSS Mexico Mexico; ^2^ Department of Pathology Oncosalud AUNA Lima Peru; ^3^ Department of Pathology Diagnostico SRL Montevideo Uruguay; ^4^ Department of Pathology IMIP Recife Brazil; ^5^ Rector de la Universidad ISALUD R.Argentina; ^6^ Department of Breast and Soft Tissue Surgery Pontificia Universidad Javeriana Bogotá Colombia

**Keywords:** breast cancer, CDx, companion diagnostics, Latin America, precision medicine

## Abstract

**Background:**

The incidence of breast cancer (BC) in LMICs has increased by more than 20% within the last decade. In areas such as Latin America (LA), addressing BC at national levels evoke discussions surrounding fragmented care, limited resources, and regulatory barriers. Precision Medicine (PM), specifically companion diagnostics (CDx), links disease diagnosis and treatment for better patient outcomes. Thus, its application may aid in overcoming these barriers.

**Recent findings:**

A panel of LA experts in fields related to BC and PM were provided with a series of relevant questions to address prior to a multi‐day conference. Within this conference, each narrative was edited by the entire group, through numerous rounds of discussion until a consensus was achieved. The panel proposes specific, realistic recommendations for implementing CDx in BC in LA and other LMIC regions. In these recommendations, the authors strived to address all barriers to the widespread use and access mentioned previously within this manuscript.

**Conclusion:**

This manuscript provides a review of the current state of CDx for BC in LA. Of most importance, the panel proposes practical and actionable recommendations for the implementation of CDx throughout the Region in order to identify the right patient at the right time for the right treatment.

## 1 INTRODUCTION

Breast cancer (BC) is the most commonly diagnosed cancer in women, with an estimated 2.1 million new cases yearly and represents 1 in 4 of all cancers in women.^1^ Over the past decade, the incidence of BC in low‐ and middle‐income countries (LMICs) has increased more than 20%. By 2020, an estimated 1.7 million new cases will occur in these countries.^2^ The higher proportion of patients diagnosed with advanced stage disease in LMICs has led to greater mortality rates compared with high‐income countries (HICs).^3^ Although significant reductions in BC mortality have been accomplished in HICs, primarily due to major investment in research and advances in early detection, treatment, and management, the same cannot be said of Latin America (LA).^3^, ^4^, ^5^

In LA, BC poses a significant public health problem and has surpassed cervical cancer as the number one cancer in women in many countries. This high‐burden malignancy represents around 200 000 new cases and more than 52 000 deaths yearly,^6^ with most cases diagnosed at locally advanced or metastatic stages when cancer care is limited. Several known risk factors that may contribute to the increase of BC incidence are changes in reproductive patterns (parity, breastfeeding), sedentary behavior, and unhealthy diet.^7^

Young women comprise 30% of LA's population, which may explain the higher prevalence of triple‐negative BC compared with other regions of the world, creating a larger burden of aggressive tumors.^8^ HICs have more disability‐adjusted life years (DALYs) for BC since they have higher general incidence rates, but the proportion of losses due to mortality are higher in LA. The mean number of DALYs lost due to BC in LA varies, being highest in Brazil, Mexico, and Argentina.^9^

In order to establish a number of recommendations in relation to precision medicine (PM) in BC care in Latin America a panel of experts in this field were provided with a series of relevant questions to address prior to a multi‐day conference. Within this conference, each narrative was edited by the entire group, through numerous rounds of discussion until a consensus was achieved.

## 2 MULTIDISCIPLINARY BC CARE

Comprehensive cancer control involves prevention, early detection, diagnosis, treatment, rehabilitation, and palliative care.^10^ In LA, most cancer care is carried out in a fragmented and sequential manner,^11^, ^12^ which contrasts with the contemporary standard of cancer care with a multidisciplinary team approach.^13^ The lack of coordination in these models is aggravated by inadequate distribution of resources. Specialized units are disproportionately located in main cities, leaving large geographic areas underserved and limiting access to innovative therapies and technologies. Furthermore, educational efforts developed for medical personnel, especially in primary healthcare, can improve diagnosis, treatment, outcomes, and increase appropriate referrals. Patient organization involvement to support patients in ensuring compliance to follow‐up care from the time of diagnosis may favor treatment continuity.

Data from LMICs, although limited, have shown the effectiveness of multidisciplinary cancer care. Evidence supports that tumor boards can aid in overcoming diagnostic and management barriers in limited‐resource settings, where specialists may be less available. An alternative to this includes partnering with regional or private academic centers to carry out remote tumor boards, allowing for increased access to multidisciplinary expertise.^14^ As molecular oncology increasingly becomes a part of standard of care, it is expected that molecular tumor boards will become as essential as disease‐specific tumor boards are today.^15^ A solution for achieving multidisciplinary care in BC is the implementation of Breast Units, which have been shown to improve overall patient outcomes.^16^ In Breast Units, the multidisciplinary management approach involves radiologists, pathologists, radiation, surgical, and medical oncologists, nurses, plastic surgeons, psychologists, geneticists, and primary care physicians, among others.^17^

## 3 BC PATIENT JOURNEY

**FIGURE 1:**
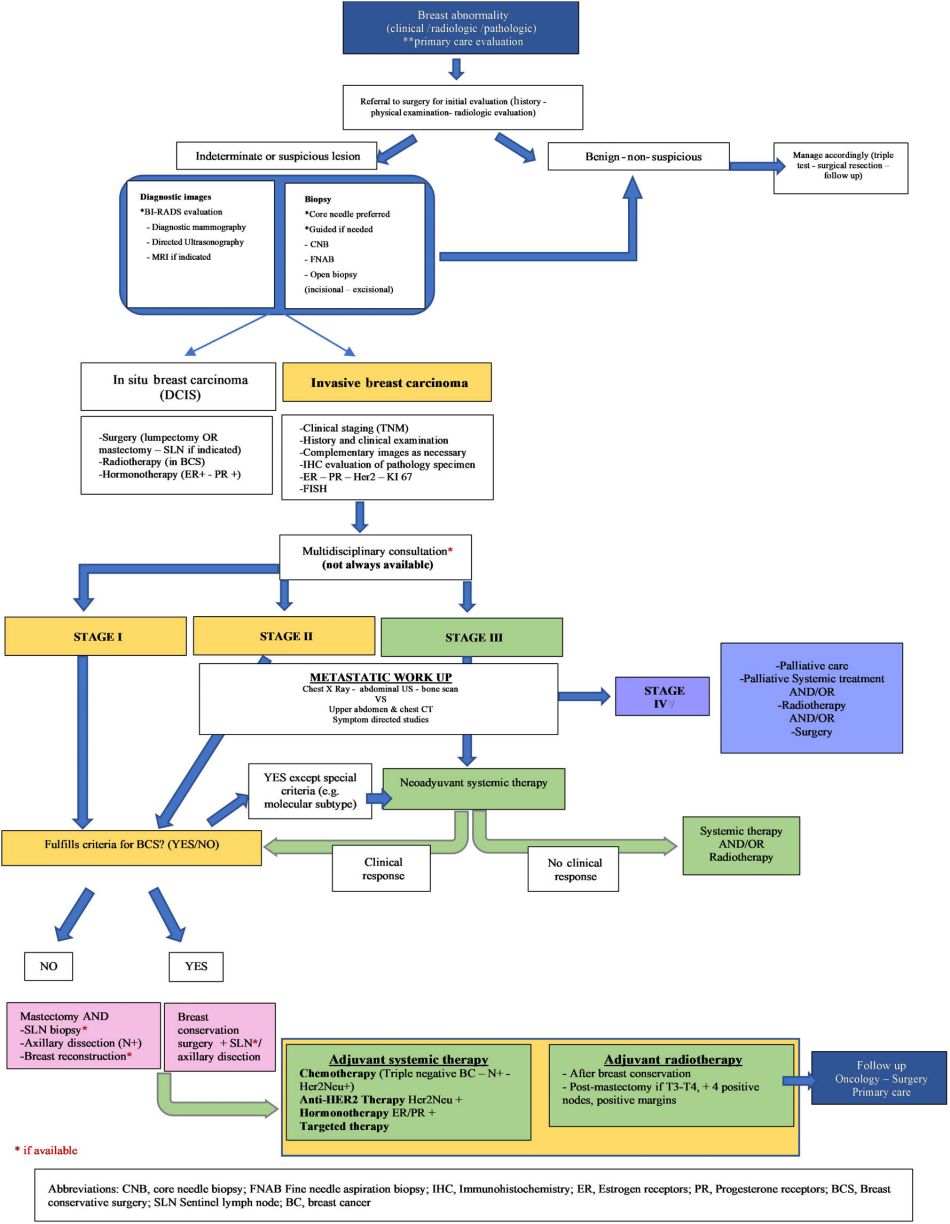
Patient journey in Latin America

In order to visualize the usual flow of relevant processes a patient undergoes when diagnosed and treated for BC in LA, a conventional process mapping of the typical patient journey is illustrated in Figure 1.

While the multidisciplinary approach is ideal, it is not available in most situations. In these cases, the patient usually starts the journey with a first consultation to a primary care doctor, followed by a surgical consultation for the evaluation of the suspicious breast condition. After the histopathologic confirmation of malignancy, a treatment plan is defined by the clinical oncologist or surgeon who then refers the patient to other professionals as needed.

In this fragmented route,^18^ coordination problems are frequent and delays in diagnosis and treatment are common. These may be due to several problems such as the absence of a structured process where patients receive support in their transit through the healthcare system and undergo proper monitoring, as well as the lack of education programs for first contact physician and patients. Frequent difficulties also exist regarding the quality of radiology and pathology reports and adherence to optimal standards of BC care. The patient journey is further complicated by reimbursement issues that obstruct access to medical care.^19^ Clinical guidelines are necessary to streamline care and could result in cost and time savings, optimal use of resources, and improvement in the quality of patient care. They are available in many countries in LA, but they are not mandatory.

## 4 IMPORTANCE OF EARLY DETECTION

Unequivocally, patients can live healthier and longer lives when provided a timely diagnosis and appropriate clinical management.^20^, ^21^ Early detection improves patient outcomes by increasing treatment options, increasing survival, and improving quality of life. The resulting diagnostic information empowers patients to better understand their prognosis and options on preventive action. Likewise, identifying genetic conditions or predispositions can be important for disease prevention and early management in patient relatives.

Cancer care varies within the different LA health systems. Financial constraints and administrative political decisions impinge cancer control, efficiency, and inclusiveness of overall cancer care. Although research on care delays and patient barriers have been conducted in select LA countries, such information has not been collated and synthesized in a standardized framework. Some reviews show common delays during every step of the cancer continuum, across cancer types, and country income‐levels.^22^, ^23^ LA women face several access barriers to standard BC care when compared to those from developed countries.^12^ This disparity is reflected in the considerable differences between the mortality and incidence ratios and the 5‐year survival rates of LMICs and HICs.^10^, ^24^

A patient with advanced stage disease poses healthcare related costs up to three to four times more than a patient with stage I disease.^10^ Evidence shows that early detection can produce “down staging” of the disease to stages that are more amenable to curative treatment and preventing early mortality.^21^, ^22^ Of note, while BC screening is an important prevention strategy, all levels of prevention should be addressed when examining solutions for better cancer care.

Mammography has remained the gold standard of BC screening throughout the world. To achieve optimal benefit, mammographic screening programs must be of high quality, appropriate targeting, and sufficient frequency.^25^ Most LA countries have some level of mammographic screening programs, with slight variations in age and intervals. In Colombia, Peru, Brazil, and Argentina, guidelines establish that mammographic screening should be performed every 2 years in women between 50 and 69 years of age, or longer depending on life expectancy. Recently in Argentina, mandatory genetic screening for BC was implemented in one province (Mendoza).

Guidelines for BC screening in Mexico recommend mammographic screening every 2 years for women starting at 40 years of age, a practice that many of the Region's medical societies recommend.^7^ Considering the high burden of aggressive tumors in young patients, healthcare systems in LA may benefit from lowering age intervals of screening programs to 40 years of age. Evidence suggests that maximum cost‐benefit can only occur if screening is done in an age group, which has a sufficiently high incidence of BC and high longevity.^26^ Comprehensive research on compliance and effectivity of screening programs for the region and the cost‐effectiveness of this practice are needed to support this notion.

## 5 RESEARCH AND CANCER REGISTRIES

The main objective of cancer registries is to collect, code, and classify cancers to produce relevant statistics and provide a framework for assessing and controlling the burden of disease. In the majority of LA, there are no regional or countrywide registries for BC and the few that exist are exclusively in the initial stages of development. This is likely a result of social, cultural, and economic barriers between and within countries that make data collection difficult and costly.^27^ An exception to this is Uruguay, which has The National Cancer Registry, a class A registry, created by law since 1989.^28^, ^29^ Establishing population‐based cancer registries (PBCRs) should be a fundamental part of any national cancer control plan to obtain high‐quality cancer data.

## 6 PERSONALIZED MEDICINE

While great advances have been made in the management and treatment of BC over the two decades, it remains a multifaceted disease exhibiting both intertumoral and intratumoral heterogeneity as well as a variable disease course. A “one size fits all” approach is in the past and the era of PM in BC is upon us.^30^, ^31^ Empowerment is found in the ability to tell payers, providers, and patients that a drug has been specifically designed for their situation. Expanding knowledge of the molecular underpinnings comprising the etiology of cancer has driven the field of PM to identify specific tumor characteristics and develop targeted therapies against these entities. The ability to predict an individual's response to a specific therapy is the ultimate goal in modern PM.^32^

### 6.1 Companion diagnostics in LA

The concept of drug‐diagnostic co‐development, or companion diagnostics (CDx), has emerged and is now the new horizon of cancer care. The FDA defines CDx as medical devices, often in vitro diagnostic devices that provide essential information regarding the safe and effective use of a corresponding therapy.^32^, ^33^ CDx are most often understood and applied in the context of specific molecular genomic findings that identify patients likely to respond to targeted therapy and stratify patients according to the molecular profile of the disease.^30^, ^32^ The success stories of trastuzumab and endocrine treatment for patients with HER2‐positive and HR‐positive BC showcase the potential of PM in cancer treatment. To date, the current list of FDA‐approved CDx remains almost exclusively based on molecular oncology targets. In BC, most are based on protein detection by immunohistochemistry (IHC).

Although CDx testing is available in LA,^13^ few studies have focused on the provision of these tests in countries that have fragmented or fragile health systems. Despite the procedural differences that may exist among the countries in this region, it is accurate to affirm that both surgical and medical oncologists request the vast majority of CDx tests in the clinical setting. In most of the Region, access to molecular testing is limited. In some countries, such as Brazil and Colombia, private and public healthcare systems only reimburse germline genetic testing ordered by certified geneticists, genetic counsellors individually, or as a part of a multidisciplinary tumor board. This imposes an additional challenge given the shortage of these specialists.

### 6.2 Benefits of CDx

CDx confer numerous benefits by identifying and delivering the right drug to the right patient at the right time. Using CDx and related pharmacotherapeutic interventions can help tackle rising health expenditures by limiting drug use to those who will benefit from the drug and reducing costs related to adverse effects. Furthermore, in some cases, targeted diagnostic approaches may be more cost efficient than performing several sequential tests.

CDx are a fundamental tool for treatment decision‐making with a significant impact in patient outcomes. They can benefit patient safety by reducing the frequency of invasive procedures that increase morbidity, limit frequency and severity of adverse effects, and improve compliance to treatment. CDx can improve the predictability of the oncology drug development process by allowing better selection of the target population, decreasing trials costs, and expediting timelines.^30^, ^31^, ^32^, ^33^ There is also added value throughout the process by providing a mechanism to collect useful data.The diverse health systems within each country, accompanied by the unique economic and regulatory stipulations require more than one financial model for CDx testing. Analyzing the vicissitudes of CDx through the lenses of all stakeholders and using investments in educational interventions are both fundamental for the consolidation of a viable model. The combination of HER2 CDx testing and trastuzumab treatment is an example of successful CDx implementation in LA. This combination is now widely covered across the Region and has evidenced significant cost benefits to healthcare systems and improved patient survival.^34^

## 7 THE ROLE OF SPECIALIZED PATHOLOGY IN BC

Selecting an appropriate diagnostic test is a challenge for clinicians, partly due to the sheer volume of choices, which is expected to increase. Additionally, contextual factors play a significant role in this decision and include treatment availability, accessibility, current standards of care, known disease characteristics, and physician experience in the use and interpretation. Collaboration between clinicians and pathologists is needed to select the right diagnostic test for right patient. Thus, pathology plays a key role in effective cancer management. However, this importance is not always reflected in the monetary investment in LA pathology departments, which often have poor infrastructure and technology, especially in the public sector.

In BC, the quality of care and clinical outcomes are directly related to the quality of breast pathology. Without accurate pathological diagnosis, clinicians may be misled, patients may receive the wrong treatment or no treatment at all, and resources may be incorrectly expended. Stakeholders must recognize breast pathology as the foundation of BC care and essential for designing any program aimed to improve the quality of care. Proper training in breast pathology for pathologists and laboratory technicians is critical and provides the underpinnings of programmatic success for any country at any level of economic wealth.^35^

## 8 MOLECULAR BIOLOGY IN CDx

The therapeutic use of molecular biomarkers relies on accurately detecting and quantifying these biomarkers for use of associated therapies. The evolution of biomarkers in BC illustrates the history of CDx and provides another perspective on how to approach current and future challenges. Although not all biomarkers are considered CDx, many function in a similar manner and provide biological, clinical, and historical context.^32^ IHC is the standard method for detecting cancer biomarkers due to its ease of use, availability, use of routine microscopy, and the ability to archive stained slides. The use of both technically and clinically unvalidated IHC assays has resulted in widespread inter‐laboratory variability in the preanalytical, analytical, and post analytical phases.^36^ In LA, IHC validation processes are not standardized, and quality control is not common practice in laboratories. ISH techniques are only available in highly specialized laboratories and institutes. The biosimilar assay development requires a long validation process that increases the cost of the assays and prolongs the delay to test availability.

## 9 THE EVOLUTION OF TARGET THERAPY IN BC

### 9.1 Multigene expression‐based assays

Identifying patients that will benefit from systemic cytotoxic chemotherapy is a persistent challenge in early BC. Although multigene expression‐based assays are not technically CDx, their importance in selecting the right patient at the right time remains. Traditionally, the decision to treat with systemic adjuvant chemotherapy was based on clinicopathological factors, as well as patient and physician preferences. However, this approach exposes a large proportion of BC patients to chemotherapy‐associated toxicity with little or no improvement in clinical outcomes. The development and commercialization of multigene expression‐based assays (eg, Oncotype DX, MammaPrint, Prosigna, etc.) have resulted in a paradigm shift in BC treatment, specifically in the care of estrogen receptor (ER) and progesterone receptor (PR) positive and HER2‐negative early stage BC.^37^, ^38^, ^39^, ^40^

### 9.2 HER2 and corresponding CDx

HER2 overexpression and the presence of abundant, accessible HER2 protein on the cellular surface in approximately 20% to 25% of BC render HER2 an ideal actionable molecule for targeted therapy.^41^, ^42^ Trastuzumab was the first FDA‐approved biologic for the treatment of HER2‐positive BC and has been shown to improve survival in 50% of the neoadjuvant, adjuvant, and metastatic settings.^43^, ^44^ Due to its high prognostic and predictive value, HER2 testing is recommended for all primary, metastatic, and recurrent BCs. Currently, there are seven FDA‐approved in vitro CDx devices to detect HER2 in BC, a prime example of how CDx leverages this relationship to design therapies for patients.^33^

For the FDA‐approved HER2 IHC CDx, different primary antibodies are used, as seen in Table 1. As with other biomarkers, HER2 IHC exhibits variability due to the inherent heterogeneity of HER2 and the inconsistencies in the processing and diagnostic phases.^45^ Lessons have been learned about the IHC platform through the HER2 Herceptin franchise, which took 5 to 7 years to optimize and resulted in a $3 billion USD loss in revenue opportunity due to IHC testing problems alone. To reduce inter‐laboratory variability, recommendations and workflows on HER2 IHC grading and scoring were published by the ASCO/CAP.^36^

**TABLE 1:** FDA‐approved in vitro CDx devices for HER2 overexpression in BC

*IHC*
HercepTest (Dako), A085 polyclonal
PATHWAY anti‐HER2/neu (Ventana Medical Systems), 4B5 rabbit monoclonal antibody
Bond Oracle HER2 IHC system (Leica Biosystems), CB11 mouse monoclonal

*ISH*
FISH assay: PathVysion HER2 DNA probe kit (Abbott Laboratories)
FISH assay: HER2 IQFISH pharmDx kit (Dako)
CISH assay: HER2 CISH pharmDx kit (Dako)
SISH/Dual ISH assay: INFORM HER2 Dual ISH DNA probe cocktail (Ventana Medical Systems)

FISH, CISH, and SISH assays quantify the *HER2/neu* gene copy number per cell using a single‐ or dual‐probe technique. Bright field ISH assays such as CISH and SISH eliminate the need for fluorescent microscopy, cost less, and are more stable over time. All FDA‐approved ISH‐based HER2 CDx employ a dual‐probe technique on paraffin‐embedded tumor samples.^46^, ^47^, ^48^ While most LA hospitals have HER2 antibody testing, the use of CDx for HER2 is limited. This trend is largely due to the high cost of the CDx test and the significantly lower cost of the regular HER2 antibody.

### 9.3 BRCA1, BRCA2, and PIK3CA

Assays for the *BRCA1* and *BRCA2* genes are currently approved as CDx for poly‐ADP ribose polymerase (PARP) inhibitors only in the context of germline mutation, which should always be accompanied by appropriate genetic counseling.^30^, ^31^, ^32^ *PIK3CA* is the most commonly mutated gene in BC and is one of the most common causes of tumor growth and endocrine treatment resistance in HR‐positive/HER2‐negative BC patients. For advanced stages of these BCs, Alpelisib, an alpha‐specific PI3K‐inhibitor used with a CDx is indicated for treatment in men and post‐menopausal women.^49^

### 9.4 PD‐L1

The era of PD‐L1, an immune‐checkpoint (IC), for BC is only beginning in LA. PD‐L1 CDx assays are increasingly available in most central pathology laboratories, but limited access remains in countries without a commercial supplier. The CDx VENTANA PD‐L1 (SP142) or DAKO PD‐L1 (22C3) assays as well as several immunotherapy drugs (eg, atezolizumab and pembrolizumab, respectively) are approved in combination with chemotherapy for women with locally advanced or metastatic triple‐negative BC and whose tumors are positive for PD‐L1.^50^ Each clone is designed for a specific drug with a different interpretation and cut‐off; therefore, clone‐specific training is required. Virtual pathology education helps facilitate training and should be implemented throughout the Region.

## 10 PITFALLS IN THE DIAGNOSTIC PHASE

In LA, approximately 37% of patients are diagnosed at an advanced stage (III‐IV).^51^ Delays between the initial suspicion and the definitive diagnosis exist in both urban and rural areas, being greater in the latter due to the centralization of these laboratories.^52^ Diagnostic decisions are further constrained by deficiencies and inequities in infrastructure, equipment, and IHC antibodies. There are limited LA studies regarding the different aspects involved in the pathologic diagnosis. Once a suspicious lesion is biopsied, the specimen goes through a process that involves pre‐analytical, analytical, and post‐analytical phases. However, many patients do not fully benefit from CDx testing because the specimen is unsuitable for testing due to poor quality control or improper transport. Rather than simply increasing testing access, stakeholders must examine other faults in the tumor specimen journey to ensure high quality and useful specimens for CDx testing.

### 10.1 Pre‐analytical phase

The pre‐analytic phase, which involves cold isquemia, fixation, and pathology request forms, is possibly the most complex phase due to the difficulty in controlling the variables that impact the quality of the specimen.

### 10.2 Fixation

Many studies have shown that fixation time impacts the quality of IHC. Prolonged fixation (>72 hours) may result in discrepancies that imply changes in the diagnostic category for HR and HER2^53^ as well as FISH signal detection.^54^ Conversely, shorter fixation times (<6 hours) may lead to immunoreactivity loss. To standardize the pre‐analytical variables, the ASCO/CAP have recommended a fixation time of 6 to 72 hours, with core needle biopsy (CNB) being on the shorter end and surgical specimens being on the longer end.^47^, ^55^

Fixation is also influenced by the concentration of fixative, pH, and buffer presence, as these factors may be responsible for masking the antigen‐binding sites, altering the three‐dimensional structure of proteins.^56^ Therefore, the use of 10% neutral buffered formalin (NBF) is recommended.^55^ In LA, the use of NBF is common practice in specialized laboratories, but not in many first level primary care hospitals, as limited commercial availability and high cost lead to using alternatives or in‐house solutions. Of note, the tissue penetration rate of the fixative agent must be adequate, especially for large samples and cases requiring long transport times, for which the specimen must be sliced at 5‐mm intervals and placed in sufficient NBF.^55^ However, this is currently not a standard practice in all care levels.

### 10.3 Cold ischemia

Cold ischemia, the time between tissue removal and fixation, is another critical step to ensure the quality of the IHC and molecular testing. The ASCO/CAP recommends a cold ischemia time <1 hour.^47^ Prolonged cold ischemia times (>1 hour) may cause proteolytic degradation and have been shown to be associated with immunogenicity loss for ER/PR,^57^ HER2,^57^ and PD‐L1.^58^ Refrigerating samples is often used in lieu of NBF in resource‐limited settings but is not best practice.

Standardizing these pre‐analytical variables in LA health systems is a challenge that requires coordination along the entire specimen pathway. Factors that contribute to this lack of standardization are: (a) absence of national protocols for tumor tissue handling that adapt the ASCO/CAP recommendations to the national realities; (b) lack of investment in pathology infrastructure; (c) poor coordination between pathology departments, operating rooms, and personnel involved in the specimen journey; and (d) lack of education about the best practices for handling specimens for all personnel involved in the process.

### 10.4 Analytical phase

In LA, the integrity of the analytical phase is compromised by a pathologist shortage, which causes excessive workloads, delayed turnaround times, and may increase human error. Thus, breast pathology training is needed for general pathologists to produce more accurate and reliable diagnoses. Finally, synoptic reports templates (provided by the CAP) are not yet standardized in LA laboratories, which is vital to care decision‐making and coordination.

Second opinion networks increase the accuracy of diagnosis, particularly for difficult cases. These networks are usually only possible through the exchange of physical slides and blocks by postal mail, which often leads to increased turnaround times and can negatively impact diagnosis and treatment. Telepathology may be useful to overcome gaps in access to formally trained breast pathologists, who are centralized in large cities. However, it has not been widely implemented in the Region. The pitfalls and potential solutions in the pathologic diagnosis of BC in LA are outlined in Table 2.

**TABLE 2:** Pitfalls in pathological diagnosis of BC and possible solutions

Gaps in Pathologic Diagnosis of BC	Suggested Solutions
Unstandardized pre‐analytical phase	Promote education about importance of pre‐analytical variables
Variable pathologist training	Improve the training curriculum Formalize BC pathology programs consider use of telepathology Involvement in clinical trial
Unstandardized pathology reports	Promote a national consensus including the main health service providers Introduce Informatics for automatization and standardize reports
Poor access to HER2 and PDL1 assays	Improve funding for BC patients
Long turnaround times	Promote the automatization on process
Few second opinion networks	Introduce telepathology networks

## 11 IMPLEMENTATION BARRIERS FOR PM

PM has demonstrated the potential to improve population health outcomes and the relative cost‐effectiveness of healthcare in other countries. Universal healthcare coverage is still lacking and ensuring sufficient public funding for BC care remains a key issue that directly impacts access to CDx.^10^ Research on access barriers and quality of care for BC is insufficient in LA.^3^ This barrier is not unique to CDx but affects the introduction of innovative therapies in general. A lack of awareness remains among all stakeholders regarding the potential system‐wide modern in cancer. The principal barriers to the widespread implementation of CDx in LA are:

1. Lack of human resources and infrastructure

PM and CDx demand an infrastructure of technological, financial, and human resources which few institutions in LA possess. Pathology departments receive inadequate funding to meet the requirements of high‐quality testing; a severe shortage of trained medical personnel exists in BC care; the existing human resources are not optimally coordinated; and multidisciplinary teams are generally unavailable in LA.

2. Availability and accessibility to targeted therapies

Not all indicated targeted therapies are commercially available in LA, and therapies currently approved are not accessible to all. In some countries, public and private payers are only required to provide select therapeutic agents, and in others, the processes of drug approval and commercialization are inefficient. Vast disparities exist in access to PM between the private and public health systems across LA, further accentuating healthcare inequities due to socioeconomic levels. Reimbursement for CDx testing in the public and private healthcare sectors has many limitations that affect optimal access in most LA countries. In countries of limited access, these tests are often paid for by pharmaceutical companies or are available to participants of research protocols. Additionally, in some countries, such as Colombia and Brazil, patients may choose the path of judicialization to access the indicated test and therapy.

## 12 FINANCIAL CONSTRAINS

Economic challenges that limit CDx implementation in the Region are mainly related to the required infrastructure and the cost of the corresponding drug. Despite the elevated upfront costs of CDx, this technology poses large potential cost savings to health systems by eliminating payments for ineffective drugs and reducing the cost associated with adverse events.^13^, ^31^ In LA, healthcare coverage disparities create a gap between patient needs and services provided, especially in the context of cancer tests and treatments that are perceived as expensive by the payers.^27^ While test costs are not prohibitive, therapeutic options are not often reimbursed, leaving patients to face exorbitant out‐of‐pocket costs.

## 13 UNSTANDARDIZED CANCER TREATMENT

Although 14 LA countries possess national cancer control plans,^59^ standardized cancer treatment protocols and guidelines within each country are sparse,^11^ which impedes CDx incorporation into clinical pathways.^27^ Well‐defined country‐specific clinical guidelines on BC management must be established, including various stakeholder perspectives and PM strategies. An informed healthcare community is vital for incorporating new technology. This shift can happen only if the diverse perspectives of patients, clinicians, healthcare leaders, and payers are integrated to create feasible and sustainable solutions.

3. Limited data

Defining metrics to measure patient outcomes and cost‐effectiveness is necessary to monitor the success of PM, of which there are no local studies in LA.^28^ Clinical utility evidence showcases the added value of a CDx to treatment management, as compared to management without. The more CDx are clinically tested, the more evidence to assure payers that these lead to overall healthcare savings. Incidence data, which is dependent on the data generated by PBCRs, are necessary for cost efficiency analyses and, ultimately, has a determining impact on public policies.^6^ In LA, most decisions on healthcare resource allocation are based solely on upfront cost, which is insufficient within systems with finite resources.^60^ Stakeholder perception of PM must pivot to appreciate the solutions that these innovative technologies can provide for the inequities of cancer care in LA.

## 14 CONCLUSIONS AND RECOMMENDATIONS

Healthcare delivery to underserved populations is challenging, but innovation in developing regions has improved the design, implementation, and financing of effective delivery models that encourage innovative therapies and may be reproduced in these areas. Many initiatives, adapted to constraints of resource‐limited environments, seek to improve initial patient contact through strengthening community roles, first‐level hospitals and primary care personnel, clinics, non‐specialized health professionals (eg, health promoters), and healthcare personnel.^61^, ^62^ In addition to indicating therapies, CDx provide unequivocal therapeutic and prognostic benefits for patients with BC. Massive data studies will continue to identify new biological targets, generate new therapies, and yield novel uses for existing therapies. The more successful CDx becomes, the more the healthcare systems must adapt to accommodate patients who may harbor multiple actionable targets, requiring multiple therapies, and multiple diagnostic modalities.

In LMICs, diagnosis and treatment are stressed by fragmented care, limited resources, and regulatory barriers. In the era of PM, where disease diagnosis and treatment can be linked for optimal results, discussions surrounding these three topics become even more complex. To facilitate cancer control, public health officials must be educated on the importance of PM's role in correctly treating the right patient at the right time. While this manuscript has focused predominantly on difficulties found in LA, these are not unique to the Region; thus, the recommendations below can be transposed to other LMICs.

GOVERNMENTS

- Develop and promote adherence to clinical guidelines in collaboration with medical societies to address fragmented care.
- Implement country‐specific protocols that adapt the ASCO/CAP recommendations to national realities for the collection, handling, transportation, and processing of tumor specimens.
- Increase availability of drugs/CDx tests and address reimbursement issues by:
  - Creating national regulatory guidelines about BC that consider various stakeholder perspectives to streamline the approval of PM strategies.
  - Optimizing dialogues between public and private sector stakeholders to develop country‐specific sustainable funding mechanisms that support shared interests and satisfy both payer and patient reimbursement needs.

HEALTH INSTITUTIONS

- Provide a *multidisciplinary approach* by creating Breast Units and molecular tumor boards adapted to the constraints of resource‐limited environments.
- Improve infrastructure by
  - Increasing investments for pathology departments to reach the level of technological and human resources required for high‐quality molecular testing with support of governmental funds and
  - Establishing laboratory accreditation programs with international standards to improve quality control.
- Increase education about the best practices for handling specimens in all the personnel involved in the process.

GOVERNMENTS, MEDICAL SOCIETIES, AND ACADEMIC INSTITUTIONS

- Address the shortage of medical personnel in the breast field by creating local training programs (pathology, surgical oncology, medical oncology, radiology, nursing, radiooncology) and implementing telepathology to overcome geographical disparity and strengthen second opinion networks.
- Implement education programs for healthcare professionals with three main focuses:
  1. Continuous medical education (CME) (programs on BC for healthcare professionals, especially in the primary care setting
  2. BC training in all specialties related to cancer care and require CME for existing specialists.
  3. Awareness within BC care specialists on innovative therapies and the testing required for them.
- Increase data collection and clinical research by:
  - Prioritizing funding and urge the importance of BC research to generate local data and define and quantify quality metrics to monitor the impact of PM.
  - Strengthening and creating PBCRs as part of regional and national cancer control plans
  - Systemically measuring treatment outcomes and conduct post‐marketing surveys with posterior development of cost‐effectiveness analysis for PM initiatives.
- Implement wide‐reaching early detection strategies by
  - Collaborating to create outreach and education programs that raise awareness among the general population on the prevention and control of BC.
  - Strengthening screening programs by considering beginning screening at 40 years of age and conducting comprehensive research on coverage, compliance, and cost‐benefit.

## CONFLICT OF INTEREST

The co‐authors declare no conflicts of interest. The organization and implementation of the consensus conference were carried out by the Americas Health Foundation (AHF), a 501(c)3 nonprofit organization dedicated to improving healthcare throughout the Latin American Region and was supported by unrestricted grants from Roche.

## AUTHOR CONTRIBUTIONS

**Isabel Alvarado‐Cabrero:** Conceptualization; formal analysis; funding acquisition; investigation; methodology; project administration; resources; supervision; validation; visualization; writing‐original draft; writing‐review & editing. **Franco Doimi:** Conceptualization; formal analysis; methodology; writing‐original draft; writing‐review & editing. **Virginia Ortega:** Conceptualization; formal analysis; methodology; writing‐original draft; writing‐review & editing. **Jurema Telles de Oliveira Lima:** Formal analysis; methodology; validation; writing‐original draft; writing‐review & editing. **Ruben Torres:** Conceptualization; methodology; visualization; writing‐original draft. **Lilian Torregrosa:** Conceptualization; formal analysis; methodology; supervision; writing‐original draft.

## ETHICS STATEMENT

In this review paper we follow the consideration published in PRISMA guidelines for systematic reviews and meta‐analyses, step by step.

## Data Availability

Data sharing is not applicable to this article as no new data were created or analyzed in this study.
